# Pain Now or Later: An Outgrowth Account of Pain-Minimization

**DOI:** 10.1371/journal.pone.0119320

**Published:** 2015-03-06

**Authors:** Hong-Yue Sun, Ai-Mei Li, Shuai Chen, Dan Zhao, Li-Lin Rao, Zhu-Yuan Liang, Shu Li

**Affiliations:** 1 Management School, Jinan University, Guangzhou, China; 2 Key Laboratory of Behavioral Science, Institute of Psychology, Chinese Academy of Sciences, Beijing, China; 3 College of Education, Shanghai Normal University, Shanghai, China; University of Pennsylvania, UNITED STATES

## Abstract

The preference for immediate negative events contradicts the minimizing loss principle given that the value of a delayed negative event is discounted by the amount of time it is delayed. However, this preference is understandable if we assume that the value of a future outcome is not restricted to the discounted utility of the outcome per se but is complemented by an anticipated negative utility assigned to an unoffered dimension, which we termed the “outgrowth.” We conducted three studies to establish the existence of the outgrowth and empirically investigated the mechanism underlying the preference for immediate negative outcomes. Study 1 used a content analysis method to examine whether the outgrowth was generated in accompaniment with the delayed negative events. The results revealed that the investigated outgrowth was composed of two elements. The first component is the anticipated negative emotions elicited by the delayed negative event, and the other is the anticipated rumination during the waiting process, in which one cannot stop thinking about the negative event. Study 2 used a follow-up investigation to examine whether people actually experienced the negative emotions they anticipated in a real situation of waiting for a delayed negative event. The results showed that the participants actually experienced a number of negative emotions when waiting for a negative event. Study 3 examined whether the existence of the outgrowth could make the minimizing loss principle work. The results showed that the difference in pain anticipation between the immediate event and the delayed event could significantly predict the timing preference of the negative event. Our findings suggest that people’s preference for experiencing negative events sooner serves to minimize the overall negative utility, which is divided into two parts: the discounted utility of the outcome itself and an anticipated negative utility assigned to the outgrowth.

## Introduction

Standard economic theory assumes that a temporal discounting process occurs when people choose between outcomes occurring at different times in an intertemporal choice. Temporal discounting refers to people’s tendency to discount the value of delayed outcomes by the amount of time one must wait for them, which leads to decreases in the subjective value of these future outcomes. The tendency to discount the value of delayed outcomes can be understood by imagining the following two options: (a) you will receive $ 100 immediately or (b) you will receive $ 100 in a week. The same absolute amounts of money received at different times are not psychologically equivalent. The subjective value of $ 100 received a week later is less than that of $ 100 received immediately.

The degree to which the value of a delayed outcome is discounted is described by many different models, including the discounted utility model [1], the generalized hyperbolic discounting model [2], the proportional discounting model [3], the quasi-hyperbolic discounting model [4], and so on. Although these models differ in their assumptions about how the value of a delayed outcome is discounted, they commonly uphold the prediction of temporal discounting that the value of a delayed outcome will be discounted by the amount of time delayed. This is the primary story told by rational economic models regarding how we should address delayed outcomes.

## When Temporal Discounting Meets Negative Events (The Waterloo)

Although there are many studies on intertemporal choices, the vast majority of these concentrate on intertemporal choices involving monetary gains and positive events rather than choices involving monetary losses and negative events. However, choices including monetary losses and negative events are equally or even more important. From an evolutionary perspective, quickly locating and flexibly responding to negative or dangerous stimuli are crucial to ensuring survival and procreation [5].

Even in the few studies exploring intertemporal choices involving negative events, a phenomenon has been identified that appears to depart from the general prediction of temporal discounting. According to the temporal discounting process, the subjective disutility of a negative event that will occur in the future is less than that of the same negative event that occurs immediately. Therefore, people prefer to experience unpleasant experiences later rather than sooner. However, immediate negative events are generally preferred to delayed negative events, casting doubts on positive time preference and showing negative time preference or negative discounting [6, 7].

Negative time preference has been found in studies examining the discounting of health outcomes. Redelmeier and Heller (1993) asked participants to rate the painfulness of several health losses that occurred at five sequentially distant times ranging from no delay to a 10-year delay [8]. The health losses were “wearing a colostomy bag for four months,” “painless bilateral blindness for four months,” and “suffering from depression for four months.” The results showed that 10% of the participants displayed a pattern of negative time preference. MacKeigan, Larson, Draugalis, Bootman, and Burns (1993) found that the fleeting health loss increased with delay [9]. Hardisty and Weber (2009) asked participants to choose between two diseases that took effect either immediately or with a delay of 1 or 10 years. The results showed that 43% of the participants exhibited zero or negative discounting [10].

Hardisty and Weber (2009) also investigated the temporal discounting of an environmental outcome [10]. They found that 30% of the participants displayed zero or negative discounting for air quality loss.

Negative time preference has also been detected in other domains. Mischel, Grusec, and Masters (1969) asked participants to indicate their preference between negative events occurring immediately and those occurring after several days/weeks [11]. They found that immediate negative events were generally preferred to delayed negative events. In their study, the negative events were eating a can of bad-tasting food, experiencing an unpleasant electric shock, drinking some very bitter liquid and experiencing a “cold presser” test. Loewenstein (1987) investigated the preference for delayed negative events using a pricing task [6]. In the study, he asked participants to name the maximum amount of money they would be willing to pay to avoid receiving a (non-lethal) 110 V shock. The shock was scheduled to occur after 5 defined delays ranging from no delay to a 10-year delay (Immediately, 3 hours, 24 hours, 3 days, 1 year and 10 years). The results showed that participants were, on average, willing to pay more to avoid a shock that was delayed than to avoid an immediate shock. This finding indicates that the participants preferred the immediate shock to the delayed shock. Berns et al. (2006) measured the neural responses when waiting for a cutaneous electric shock [12]. They found that some individuals chose to receive electric shock earlier rather than later, even at the cost of receiving more voltage. Harris (2012) examined the participants’ time preferences for different types of negative events including social rejection, embarrassment, pain and monetary or property loss [13]. She found that many participants preferred to experience unpleasant experiences sooner rather than later in all types of negative events except for the monetary or property loss.

In summary, if the intertemporal choice of negative events is studied purely in the frame of the temporal discounting process, the explanation and prediction of observed behavior are at a deadlock because the disutility assigned to the delayed option (later pain) should not be greater than that assigned to the immediate option (sooner pain). Furthermore, the preference for the immediate negative event would contradict the principle of maximizing individual interests assumed by mainstream economic and decision theories [14, 15].

## Possible Accounts

If choosing a dominant option is not a decision bias or error, how can the deadlock be resolved? Going beyond the frame of temporal discounting process, Loewenstein (1987) assumed that the anticipation of future events impacted the choice [6]. Specifically, he speculated that participants might factor in an anticipated dread toward the delayed negative event that made the delayed negative event more aversive than the immediate negative event.

Based on the dread-based interpretation of Loewenstein (1987) [6], we could potentially better understand the anomalous preference by taking the perspective of modifying a representation space. In classical decision theory, decision making can be regarded as an outcome of mental processes leading to the selection of a course of action from among several options [16], with each offered option being characterized by a given set of dimensions (or attributes) that presumably remain unchanged for the mental processes. On the premise that such a representation space remains unchanged in the decision processes, the choices are presumably guided by the principle of value maximization [14]. However, in recent years, researchers have started to explain some seemingly unusual behaviors from the perspective of modifying a representation space. A recent fMRI study probing the neural basis of superstition [17] provides supportive evidence that some paradoxical choices can be explained if the decision maker’s final decision is based on an unoffered dimension/attribute, i.e., based on auspiciousness rather than on an offered monetary dimension. If an intertemporal choice between negative events is restricted to the offered outcome and various time attributes, the preference for an immediate outcome is a bias or error. However, such a preference is explicable if the utility of the unchosen option is assigned not only to the offered outcomes but also includes something unoffered, which we termed the “*outgrowth*”.

We hypothesize that the proposed outgrowth accompanying the delayed negative events is mainly represented by the anticipated emotional distress toward the delayed negative events. The anticipated emotional distress has two components. The first component is the actual experience of the anticipated emotional distress, that is, the anticipated negative emotions elicited by the delayed negative event, such as anxiety, fear and dread, as discussed by Loewenstein [6]. The other component is the utilitarian impact the emotional distress will have on the life of an individual, which we call “the anticipated rumination.” The rumination denotes that one cannot stop thinking about the negative event during the waiting process. Related studies [18, 19] on one’s experience during a waiting period provide supporting evidence that persistent and repetitive thoughts associated with rumination are likely to arise when awaiting an uncertain outcome (not a negative event in the present study). Moreover, rumination could increase one’s attention to the future event and enhance the intensity of the inherent negative emotions elicited by the event [18, 20]. The anticipated emotions and the anticipated rumination can work together to reflect a negative anticipation of the future, from which a negative utility is derived, thus making the delayed option more aversive.

With the existence of the outgrowth, the deadlock alluded to above can be easily resolved if we assume that the value of a future outcome is not restricted to the discounted utility of the outcome per se but is complemented by a anticipated negative utility assigned to the outgrowth. We supposed that decision-makers would still act as profit maximizers when faced with an intertemporal choice between negative events given that the unchosen option would be more painful. According to this explanation, decision makers continue to optimize the utility of a future outcome regardless of whether they face a gain (positive event) or a loss (negative event).

To date, no previous research has examined the existence of “dread” or the existence of what we call the “outgrowth”. Although Harris (2012) believed that her results supported the dread-based interpretation, she appeared to infer this conclusion from the decision outcome [13]. She provided no direct evidence for the existence of the anticipated dread. Therefore, the aim of this study is to empirically investigate the mechanism underlying the preference for immediate negative outcomes. Study 1 was conducted to examine whether the outgrowth was generated in accompaniment with the delayed negative events. Study 2 was conducted to examine whether people actually experienced the negative emotions they anticipated in a real situation of waiting for a delayed negative event. Study 3 was conducted to test whether the intertemporal choice between negative events can be explained and predicted by the pain-minimization principle with the help of the outgrowth.

## Study 1: Does the Outgrowth Exist? A Content Analysis

According to the reason-based analysis [21], when faced with a choice, decision makers often construct reasons to resolve a conflict and justify their choices. Therefore, in Study 1, we applied content analysis to analyze the reasons for the timing preference for negative events to examine whether an outgrowth was actually generated regarding the delayed negative events. Instead of investigating the timing preference for an unusual negative event, such as the electric shock used in Loewenstein (1987) [6], we chose to explore preferences for the timing of stressful events common in our daily lives. Study 1a explored when the participants would choose to undergo an operation, and Study 1b investigated whether the participants were willing to postpone an exam.

## Study 1a: When Do People Want to Undergo an Operation?

### Participants

The participants were 77 undergraduates (53 women and 24 men) from Jinan University. The mean age of the participants was 23.31 years (*SD* = 4.12, range = 20–25). The participants were recruited for the experiment via posters put up around campus, a bulletin board system, and the students’ online communities. Each participant was given ¥15 for their participation and cooperation. The study was approved by the Institutional Review Board of the Institute of Psychology, the Chinese Academy of Sciences. Because the data were analyzed anonymously, and no apparent ethical research complication with participation could be identified, informed oral consent was recommended and obtained from participants before data collection. Participants were given the opportunity to refuse to participate, to omit questions or to withdraw from the study at any time without penalization. This procedure was supervised by two experimenters and was documented by an experimenter.

### Procedure and materials

A computer presented the participants with a hypothetical situation in which they would have to undergo a painful operation. The curative effect did not vary regardless of when the operation was performed, and the disease would not worsen. The participants were asked to choose from among five alternative times to undergo the operation: *right now*, *2 hours from now*, *1 day from now*, *1 week from now* and *1 month from now*. We then interviewed the participants about the reasons for their choice. The participants also recorded each reason on the computer.

### Results and discussion

#### The choice

**Fig 1:**
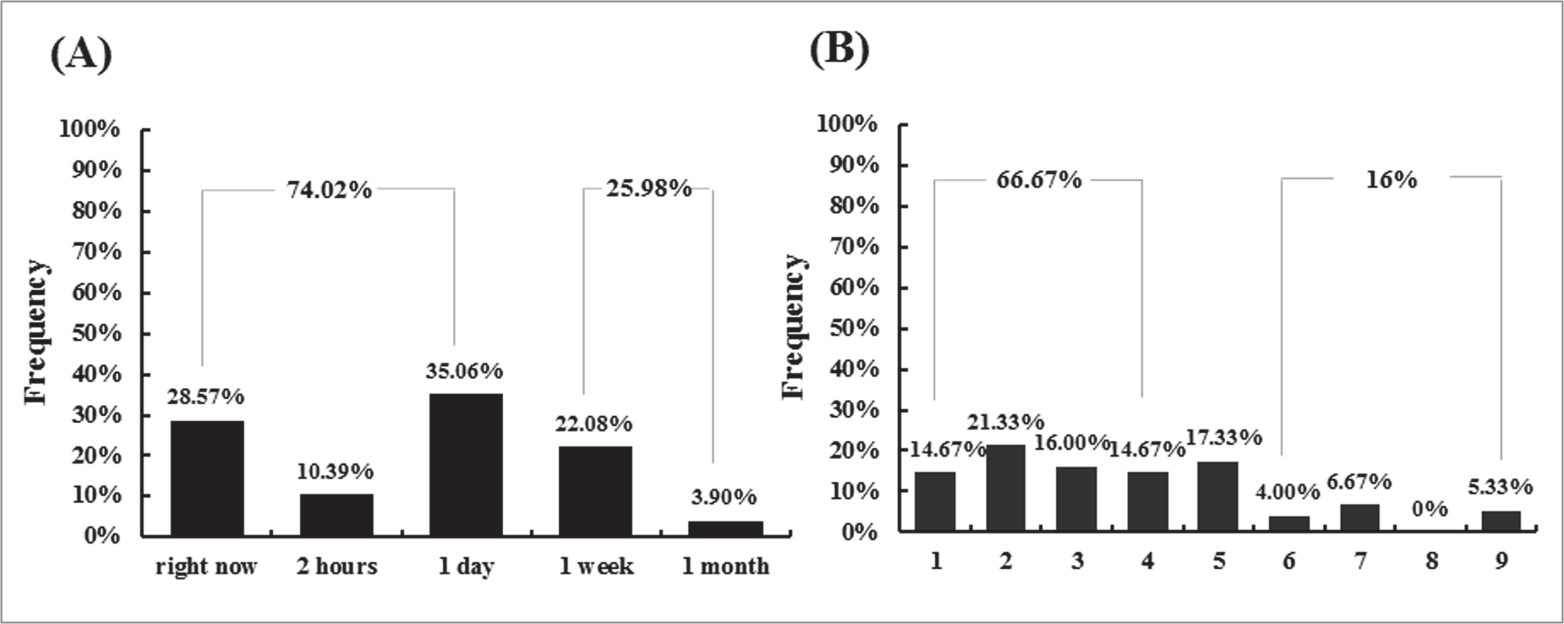
Panel A: The proportion of participants who chose each of the 5 available times at which they wanted to undergo an operation from Study 1a. Panel B: The proportion of participants who chose each of the 9 available rating points to expressed their willingness to delay the exam in Study 1b (1 = not at all willing, 9 = totally willing).

As Fig. 1A illustrates, most participants preferred to undergo an operation immediately (28.57%) or after a one day delay (35.06%), and fewer than 4% of the participants chose to undergo an operation after a one-week delay. For comparison, we divided the participants into two groups according to their choice: participants who chose to undergo an operation within a day (e.g., right now, 2 hours from now, and 1 day from now) and those who chose to undergo an operation after a day (e.g., 1 week from now and 1 month from now). A chi-square test showed that significantly more participants (74.02%) wanted to undergo an operation within a day rather than after a day (25.98%), χ^2^ (1, 77) = 17.78, *p*<0.001.

#### Reasons listed

The participants listed 160 reasons for their choices, with an average of 2.08 reasons per participant (*SD* = 0.59). Of these, 15 were considered to be unrelated to the task and excluded from the analysis.

A code table consisting of six reasons was formed based on a preliminary interview. Two independent coders coded each reason by identifying whether the primary topic of the reason was associated with one of the six reasons in Table 1. The inter-rater reliability was 0.92. The first three reasons supported undergoing an operation early. The reason “The delayed operation is a burden that would make me anxious and stressed” reflected the anticipated negative emotions elicited by the delayed operation, and “I will get distracted from other things by the delayed operation” reflected the anticipated rumination during the waiting process, in which one cannot stop thinking about the negative event. These two reasons were assumed to constitute the elements of the outgrowth. The reason “I want to get rid of the disease and recover as soon as possible” reflected the desire for recovery from a sustainable disease. Three reasons supported undergoing an operation later: making preparations, “My family and I could prepare for the operation mentally and physically”; avoidance, “Because the operation is painful, I want to put it off as long as possible”; and being afraid of an accident, “I want to put it off as long as possible because I am worried about an accident during the operation.”

**Table 1:** The summary and comparison (the two-sample t test) of the proportion of reasons listed by participants who preferred to take the operation within a day and those who preferred to take the operation after a day in Study 1a.

Reasons	Makes people prefer to take the operation…	within a day	after a day	*t*
1. I want to get rid of the disease and recover as soon as possible. (desire for recovery)		0.32	0.03	6.15***
2. I will get distracted from other things by the delayed operation (outgrowth: the anticipated rumination).	Early	0.09	0	3.62***
3. The delayed operation is a burden that would make me anxious and stressed (outgrowth: the anticipated negative emotions).		0.40	0	7.90***
4. My family and I could prepare for the operation mentally and physically. (making preparations)		0.19	0.70	-5.10***
5. Because the operation is painful, I want to put it off as long as possible. (avoidance)	Later	0	0.14	-1.71
6. I want to put it off as long as possible because I am worried about an accident during the operation. (being afraid of the accident)		0	0.12	-1.37

*p<.05.

**P<.01.

***P<.001

The analysis of reasons followed a method applied in Hardisty, Appelt, and Weber (2012) [22]. For each category of reason in the code table, we used the averaged proportion of the specific reasons participants gave as a measure of their relative prevalence. For example, if one participant listed a total of two reasons that fell within two different categories, the proportion for each category of reason was coded as 0.5 and the proportion for the other four categories was coded as 0. If both reasons fell within the same category, the proportion for that category of reason was coded as 1, and the proportion for the other five categories was coded as 0. If one participant listed three reasons that fell within three different categories, the proportion for each category of reason was coded as 0.33, and the proportion for the other three categories was coded as 0. If two of the three reasons fell within the same category and the other reason fell within another category, the proportions for the former, the latter, and all other categories were coded as 0.67, 0.33 and 0, respectively. In this way, each participant had a proportion distribution for six categories of reason. Finally, for each category of reason, we averaged the proportions computed for different participants. Therefore, each category of reason resulted in an average proportion, which was used as a measure of the relative prevalence of each category of reason.

As Table 1 illustrates, the two-sample t test results showed that for each of the reasons supporting undergoing an operation early, a significant difference was found between the participants who preferred to undergo an operation within a day and those who preferred to undergo an operation after a day. The participants who preferred to undergo an operation within a day listed a significantly larger proportion of reasons related to the outgrowth (*M* = 0.09 for reasons concerning the anticipated rumination and *M* = 0.40 for reasons concerning the anticipated negative emotions) than those who opted to undergo an operation after a day (*M* = 0 and *M* = 0, respectively), *t*_*anticipated rumination*_ (76) = 3.62, *p*<0.001, *t*_*anticipated negative emotions*_ (76) = 7.90, *p*<0.001. Among the reasons supporting undergoing an operation later, only making preparations showed a significant difference between the participants who preferred to undergo the operation within a day and those who preferred to undergo the operation after a day, *t*_*making preparations*_ (76) = -5.10, *p*<0.001.

In Study 1a, as we predicted, people actually generated an outgrowth toward the delayed negative operation, which prevented them from waiting. The results showed that the participants who preferred to undergo an operation earlier listed more reasons related to the outgrowth than those who chose to delay the operation. Furthermore, the outgrowth was composed of two elements. The first component is the anticipated negative emotions elicited by the delayed operation, and the second is the anticipated rumination elicited by waiting.

However, we hypothesized that the operation scenario may mask or attenuate the effect of the outgrowth on decision behavior due to the continuous nature of the disease before the operation. As the results showed, people who preferred to undergo the operation quickly listed a large proportion of reasons related to their wish to be rid of the disease. To overcome these issues, we conducted Study 1b.

## Study 1b: Are People Willing to Postpone an Exam?

In Study 1b, we chose to explore the timing preference for another common stressful event, an exam, to obtain more reliable results. Unlike the operation scenario, the exam scenario was not related to a continuous disease. In this study, we asked the participants to rate their willingness to have an exam postponed rather than choosing the timing for it from among multiple-alternative options.

### Participants

A total of 75 undergraduates (51 women and 24 men) from Jinan University enrolled in the study. The mean age of the participants was 22.44 years (*SD* = 1.74, range = 19–27). They were recruited for the experiment via posters put up around the campus, a bulletin board system, and the students’ online communities. Each participant was given ¥15 for their participation and cooperation. The study was approved by the Institutional Review Board of the Institute of Psychology, the Chinese Academy of Sciences. Because the data were analyzed anonymously, and no apparent ethical research complication with participation could be identified, informed oral consent was recommended and obtained from participants before data collection. Participants were given the opportunity to refuse to participate, to omit questions or to withdraw from the study at any time without penalization. This procedure was supervised by two experimenters and was documented by an experimenter.

### Procedure and materials

Each participant considered an exam delay situation. The scenario was presented on the computer and read as follows: *Imagine that you have been preparing to take an exam tomorrow*. *However*, *you have been notified that the exam could be postponed until three days later*. The participants were asked to rate their willingness to have the exam delayed on a 9-point scale ranging from 1 (not at all willing) to 9 (totally willing). The experimenter then interviewed each participant about the reasons for their rating results. The participants recorded their reasons on the computer.

### Results and discussion

#### Willingness

For comparison, we divided the participants into three groups: *unwilling to delay* (ratings scores from 1 to 4), *uncertain whether to delay* (rating scores of 5) and *willing to delay* (ratings scores from 6 to 9). The rating result is shown in Fig. 1B. A chi-square test showed that there were significantly more participants (66.7%) who were unwilling to delay the exam than who were uncertain as to whether to delay the exam (17.3%) or willing delay the exam (16%), χ^2^ (2, 75) = 37.52, *p* < 0.001.

#### Reasons listed

One participant failed to record his reasons on the computer and subsequently his data were excluded. The participants listed a total of 147 reasons for their choices, and there was an average of 1.98 reasons (*SD* = 0.65) per participant. Of these, 19 reasons were considered to be unrelated and were excluded from the analysis.

A code table was formed based on a preliminary interview. Two independent coders coded each reason by identifying whether the primary topic of the reason was associated with one of the reasons shown in Table 2. The inter-rater reliability was 0.90. The first four reasons supported not delaying an exam. “The delayed exam is a burden that would make me anxious and stressed” represented the anticipated negative emotions elicited by the delayed exam, and “I will get distracted from other things by the delayed exam” reflected the anticipated rumination elicited by the waiting process. These two reasons were assumed to be the primary elements of the outgrowth. “Because I am well prepared for the exam, no extra preparation is necessary” reflected the idea of not needing further preparation, and “I am worried that there would be some changes in 3 days, for instance, forgetting what I reviewed, others making better preparations, and so on” reflected uncertainty about the future. “I could further prepare for the exam” reflected the motivation of being more thoroughly prepared, and “Because I have prepared sufficiently, it does not matter when the exam begins” reflected indifference. These two reasons supported delaying the exam. Like Study 1a, for each category of reason in the code table, we used the averaged proportion of the specific reasons participants gave as a measure of their relative prevalence.

**Table 2:** The summary and comparison (MANOVA) of the proportion of reasons listed by participants in three groups: unwilling to delay, uncertain as to whether to delay, and willing to delay in Study 1b.

Reasons	Makes people prefer to take the exam…	unwilling to delay	uncertain whether to delay	willing to delay	*F* (η^2^)
1. Because I am well prepared for the exam, no extra preparation is necessary. (no need for further preparation)	Early	0.14	0	0	3.49*(0.09)
2. I will get distracted from other things by the delayed exam (outgrowth: the anticipated rumination)		0.26	0.08	0.05	3.16* (0.08)
3. The delayed exam is a burden that would make me anxious and stressed (outgrowth: the anticipated negative emotions)		0.21	0.04	0	4.92**(0.12)
4. I am worried that there would be some changes in 3 days, for instance, forgetting what I reviewed, others making better preparations, and so on. (uncertainty of future)		0.29	0.19	0.05	2.63(0.07)
5. I could further prepare for the exam. (further preparation)	Later	0.04	0.31	0.41	12.12*** (0.25)
6. Because I have prepared sufficiently, it does not matter when the exam begins (indifferent attitude)		0.04	0.38	0.50	18.94*** (0.35)

*p<.05.

**P<.01.

***P<.001.

As illustrated in Table 2, the MANOVA test revealed that, except for “uncertainty of future”, the proportion of each of the reasons was significantly different among the three groups of participants. The participants who were unwilling to delay an exam listed significantly higher proportions of reasons concerning the outgrowth than those who were uncertain or willing to delay an exam, *F*_*anticipated rumination*_ (2, 71) = 3.16, *p* = 0.049, η^2^ = 0.08; *F*_*anticipated negative emotions*_ (2, 71) = 4.92, *p* = 0.01, η^2^ = 0.12.

In study 1b, we adopted the delayed exam scenario to explore the timing preference of the participants for a delayed negative event. To overcome the deficiency of the operation scenario in Study 1a, the exam scenario did not involve the similar issue of preferring to undergo an operation quickly to be rid of a continuous disease. In such a scenario, we still found that the outgrowth toward the delayed exam prevented people from wanting to postpone the exam. The results showed that the participants who chose to take an exam earlier listed more reasons related to the anticipated negative emotions and the anticipated rumination than those who opted to delay the exam.

The overall results of Study 1 indicated that the outgrowth in delayed negative events was present and that the outgrowth was primarily reflected as two elements in favor of the delayed operation and exam. One element was the anticipated negative emotions elicited by the delayed negative event, and the other was the anticipated rumination elicited by the waiting, in which one cannot stop thinking about the negative event.

## Study 2: Is the Outgrowth Experienced during the Waiting Process? A Follow-Up Investigation

The results of Study 1 indicated that the outgrowth was indeed generated in conjunction with the decision to delay the negative event. Previous studies have shown that the predicted and online experiences of individuals are often inconsistent. The intensity of predicted experiences is usually stronger than online experiences. For example, Mitchell, Thompson, Peterson, and Cronk (1997) found that the predicted experiences of individuals concerning meaningful life events are more positive than their online experiences [23]. Wirtz, Kruger, Scollon, and Diener (2003) also found that students’ predicted experiences of a spring break are either more positive or more negative than online experiences [24]. Therefore, we are interested in examining whether individuals actually experienced the negative emotions they anticipated. If not, choosing an immediate negative event due to the anticipated negative emotions could be a type of decision bias. Study 2 aimed to answer this question. In this study, we conducted a follow-up investigation to examine whether the anticipated negative emotions are actually experienced in a real situation of waiting for a delayed negative event.

### Participants

The participants were 31 postgraduates majoring in psychology (22 women and 9 men) from Jinan University who agreed to participate for extra academic credit. The mean age of the participants was 25.19 years old (*SD* = 0.60, range = 24–26). The study was approved by the Institutional Review Board of the Institute of Psychology, the Chinese Academy of Sciences. Because the data were analyzed anonymously, and no apparent ethical research complication with participation could be identified, informed oral consent was recommended and obtained from participants before data collection. Participants were given the opportunity to refuse to participate, to omit questions or to withdraw from the study at any time without penalization. This procedure was supervised by two experimenters and was documented by an experimenter.

### Procedure and materials

The data were collected before the final examination of an organizational behavior course at the end of the semester in 2012. One of the assessment assignments was a 10-minute PowerPoint presentation. The participants had to choose one topic they were interested in from the top journals in either management science or psychology. The presentation was scheduled to occur 14 days later (4 January, 2013), and the score would account for 80% of the total grade of the organizational behavior course.

Participants received a text message at 21:30 every evening from 21 December, 2012, to 3 January, 2013, that asked them to complete a 9-point Likert scale in electronic form for 6 anticipated emotions (see S1 Appendix). Based on the interview conducted in Study 1, we chose to measure four anticipated negative emotions: *anxious*, *worried*, *stressed*, and *afraid*. A positive emotion and an irrelevant negative emotion were also measured: *happy* and *angry*, respectively. The completed scale was emailed back to the experimenter the same evening. On the presentation day (4 January, 2013), the participants completed the scale for the last time at 12:30. The presentation started at 13:30.

### Results and discussion

**Fig 2:**
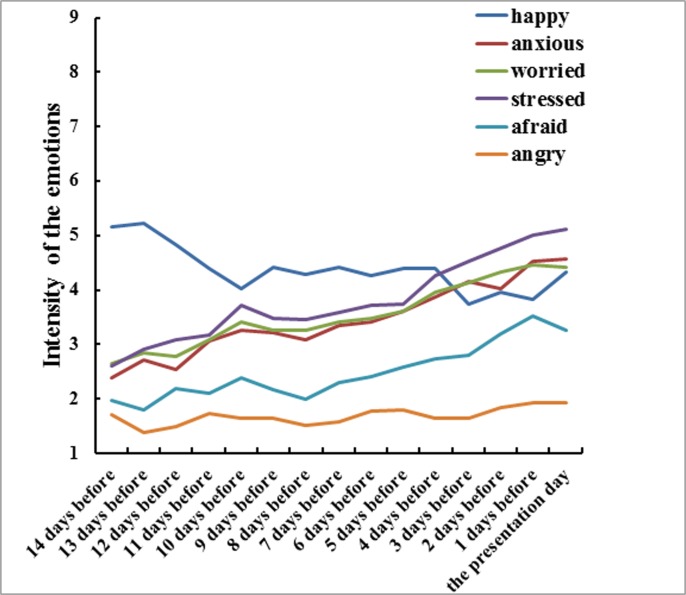
The time course of 6 anticipated emotions before the presentation in Study 2.

Six repeated measures ANOVA analyses were conducted separately to compare the intensity of each anticipated emotion among the different time points before the presentation. As Fig. 2 illustrates, as the presentation approached, the intensity of *happy* decreased gradually: *F*_*happy*_ (5.12, 153.53) = 2.81, *p* = 0.018, η^2^ = 0.09; the intensities of *anxious*, *worried*, *stressed*, *afraid* increased gradually: *F*_*anxious*_ (5.59, 167.72) = 10.19, *p* < 0.001, η^2^ = 0.25; *F*_*worried*_ (4.13, 123.81) = 7.13, *p* < 0.001, η^2^ = 0.19; *F*_*stressed*_ (3.97, 119.06) = 10.13, *p* < 0.001, η^2^ = 0.25; and *F*_*afraid*_ (4.74, 142.04) = 6.84, *p* < 0.001, η^2^ = 0.19. The intensity of *angry* did not obviously rise or decline, *F*_*angry*_ (4.49, 134.68) = 1.01, *p* = 0.412, η^2^ = 0.03.

The results of Study 2 showed that the participants actually experienced a number of anticipated negative emotions they anticipated when waiting for a negative event. More importantly, the intensities of the anticipated negative emotions gradually increased along with the temporal proximity to the upcoming negative event.

## Study 3: Could the Existence of the Outgrowth Make the Minimizing Loss Principle Work?

The combined results of Study 1 and Study 2 suggested that the outgrowth actually accompanied a delayed negative event. Study 3 aimed to explore how people finally make a choice when faced with an immediate and a delayed negative event. Specifically, we wanted to examine whether the existence of the outgrowth could make the maximization principle work. Furthermore, in this study, we not only explored the choice wherein one of the options was immediately available, but we also explored the situation wherein both options lay in the future.

### Participants

A total of 256 undergraduates (176 women and 80 men) from Jinan University enrolled in the study. The mean age of the participants was 20.83 years (*SD* = 1.99, range = 17–34). The participants were recruited for the experiment via posters put up around the campus and were given a small gift for their participation and cooperation. The study was approved by the Institutional Review Board of the Institute of Psychology, the Chinese Academy of Sciences. Because the data were analyzed anonymously, and no apparent ethical research complication with participation could be identified, informed oral consent was recommended and obtained from participants before data collection. Participants were given the opportunity to refuse to participate, to omit questions or to withdraw from the study at any time without penalization. This procedure was supervised by three experimenters and was documented by an experimenter.

### Procedure and materials

The participants were randomly allocated to one of the two groups. In the first group, the participants were asked to imagine that they were going to receive a 40 V electric shock and to answer three questions: (1) “when would you prefer to receive the electric shock?” (Option A: right now; Option B: 1 week from now). We labeled this condition the *imminent future choice condition*. The participants were then asked to indicate the strength of their commitment to the selected option on a 6-point scale (1 = very sure of choice A, 6 = very sure of choice B). (2) “How much pain (mental and physical) in total do you anticipate feeling if the electric shock happens right now (option A)?” (3) “How much pain (mental and physical) in total do you anticipate feeling if the electric shock happens 1 week from now (option B)?” The participants were given a 128 mm line with end-points labeled ‘no pain’ on the left and ‘excruciating pain’ on the right. They were asked to mark the line to indicate the intensity of the pain that they anticipated the electric shock would cause them now or later. The distance from the left end of the scale to each participant’s mark was measured and used as the indicator of pain anticipation.

The participants in the second group were asked the same three questions as the participants in the first group except that the time options for the electric shock were different. The participants were asked when they would prefer to receive the electric shock (option A: 1 week from now; option B: 2 weeks from now). We labeled this condition *the remote future choice condition*. In both groups, the order of the three questions was counterbalanced. Approximately half of the participants made their choice first and the other half completed the measurement of pain anticipation first.

### Results and discussion

#### Choice

A 2 (question order: choice first vs. choice last) x 2 (condition: immediate future vs. remote future) ANOVA was performed to examine the effects of the question order and condition on choice. The results showed that neither of these two factors had a significant main effect on choice (*F*_*question order*_ (1, 251) = 0.04, *p* = 0.84, η^2^ < 0.001; *F*_*condition*_ (1, 251) = 0.27, *p* = 0.60, η^2^ = 0.001), and there was no significant interaction between these two factors (*F*_*interaction*_ (1, 251) = 3.20, *p* = 0.08, η^2^ = 0.013). The results indicated that neither the question order nor the condition significantly affected the choice. Therefore, we combined the choice data for the two question orders and the two conditions for further analysis. The mean rating for choice was 2.00 (*SD* = 1.19), suggesting that people have a preference for experiencing pain sooner, regardless of whether the choice sets involved an immediate option.

#### Pain anticipation

**Fig 3:**
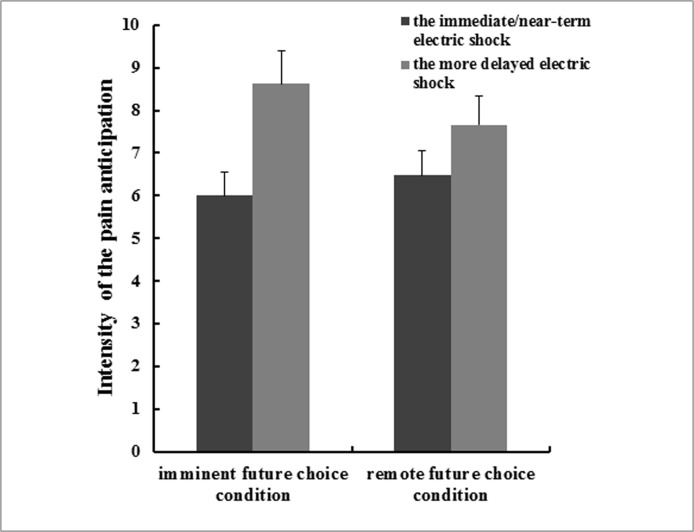
The average pain anticipation for the immediate/near-term electric shock and the more delayed electric shock in Study 3.

In the imminent future choice condition, 82.1% of the participants reported that the immediate shock would hurt them more deeply than the delayed shock. As Fig. 3 shows, the participants anticipated more pain for the electric shock occurring 1 week later (*M* = 8.62, *SD* = 2.91) than for the shock occurring right now (*M* = 6.00, *SD* = 2.89), *t* (122) = -1.98, *P*<0.001. A linear regression was performed to predict the rating of choice, with the difference in pain anticipation between these two electric shocks occurring at different times (pain anticipation for an electric shock occurring 1 week later minus that for an electric shock occurring right now) entered as the independent variable. The results showed that the difference in pain anticipation could significantly predict the rating of choice, *beta* = -0.21, *P*<0.001. That is, if the pain anticipation at the electric shock occurring 1 week later was larger than that for the electric shock occurring right now, the participants would prefer to experience the shock immediately; otherwise, they chose to delay the electric shock. Similar results were found for the remote future choice condition. Of the total participants, 73.7% reported that the immediate shock would hurt them more deeply than the delayed shock. The participants anticipated more pain for the electric shock occurring 2 weeks later (*M* = 7.66, *SD* = 2.70) than for the shock occurring 1 week later (*M* = 6.48, *SD* = 2.55) (see Fig. 3), *t* (132) = -5.08, *P*<0.001. A linear regression showed that the difference in pain anticipation could significantly predict the rating of choice, *beta* = -0.18, *P*<0.001.

The results of Study 3 showed that participants anticipated more pain in total if the electric shock lay in the future and that the difference in pain anticipation between the immediate/ near-term shock and the delayed shock could significantly predict the timing preference for the electric shock.

## General Discussion

Previous studies that explored the preference for the timing of various negative events have found a preference toward experiencing negative events sooner rather than later [6, 8–13]. The results reported in this research add evidence to the findings. We found that most participants preferred to undergo an operation within a day, preferred to make a presentation earlier and were not willing to postpone an exam. The previous findings and our results indicated that such a preference pattern is a global phenomenon in intertemporal choice confirmed by various decision forms including a binary-alternative decision structure, a multiple-alternative decision structure, a pricing task and a rating task.

From the perspective of modifying a representation space, we believe that the value of the delayed outcome in the intertemporal choice of negative events is assigned not only to offered dimensions (or attributes) but also to unoffered dimensions, which we termed the “*outgrowth*.” We provided evidence to support the existence of the outgrowth using a content analysis. In Study 1, we found that the average proportion of reasons addressing the outgrowth accounted for approximately 50% (49% in Study 1a and 47% in Study 1b) of all of the reasons listed by participants who preferred to experience the operation and exam earlier. We also found in Study 1 that the outgrowth toward the delayed negative events prevented people from wanting to postpone negative events. The participants who preferred to experience the negative events earlier listed more reasons related to the outgrowth than those who opted to delay the negative events.

Furthermore, we presumed and demonstrated that the investigated outgrowth was reflected as two elements. One was the anticipated negative emotions elicited by the delayed negative event, and the other was the anticipated rumination during the waiting process in which one cannot stop thinking about the negative event. This finding extended the anticipated dread interpretation demonstrated by Lowenstein (1987) [6]. The anticipated negative emotions accounted for a large proportion of the total reasons for both the operation and exam events. Therefore, we speculated that anticipated negative emotions might be the most universal and crucial element of the outgrowth. More importantly, the contribution made by the anticipated rumination in the decision process varied for the operation and exam events. The participants facing a delayed exam were more worried that they would always be thinking about the delayed event and would thus distract them from other scheduled events compared with those facing a delayed operation. We therefore conjectured that the existence and intensity of the anticipated rumination might be different for different negative events.

Using a follow-up investigation in Study 2, we found that participants actually experienced a number of negative emotions related to the temporal proximity to the upcoming class presentation. The combined results of Study 1 and Study 2 suggested that the predictions of individuals regarding their possible emotional distress during the waiting process are consistent with their online experience. Therefore, this excludes the possibility that choosing an immediate negative event is a type of decision bias, at least on this point.

Next, we supported the claim that choosing an immediate negative experience is a decision reached by following the principle of maximizing individual interests. In Study 3, participants anticipated that they would feel more pain from the electric shock in the future, and the difference in pain anticipation between the immediate option and the delayed option could significantly predict the timing preference for the electric shock. The findings supported our assumption that people’s preference for experiencing negative events sooner serves to minimize the overall negative utility, which is divided into two parts: the discounted utility of the outcome itself and an anticipated negative utility assigned to the outgrowth.

## Theoretical implications

Breaching the confinement of the mainstream discounted utility models, the present paper explored the psychological mechanism of the negative discounting phenomenon from the perspective of modifying a representation space. As our findings about the outgrowth implied, decision-makers do not passively choose between options by relying on an offered set of dimensions but actively generate an extra underlying dimension and assign a delayed utility to the underlying dimension as an “outgrowth.” The utility of the chosen option is assigned to include not only something that is offered but also something unoffered.

The breakthrough of the conventional idea of representing an option in the given dimensions can shed light on model building and revision in decision making. The decision analysis developed by von Neumann and Morgenstern in the 1940s was applied to a well-formed multi-dimensional representation space without altering the given set of dimensions (or attributes). Most current risky and intertemporal choice models are also developed on the premise that such a representation space remains unchanged in the decision process. With the modification of the representation space, we can also discover explanations for some seemingly unusual behaviors.

The present results cast doubt on the intertemporal choice models, which only represent the options on the two given dimensions of time delay and outcome, including the mainstream discounted utility models and some non-mainstream models. This paper established a foundation for further constructing and revising models that could well explain the intertemporal choice behaviors in the loss domain. Integrating motivation, emotions, and other factors with the current models or integrating different models is a good way to describe human behaviors. For example, Steel and König (2006) constructed a temporal motivational theory based on the fundamental features of picoeconomics, expectancy theory, cumulative prospect theory, and need theory to better understand human behavior [25].

## Future Directions and Implications

As the results of Study 3 suggested, most participants (82.1% in the imminent future choice condition and 73.7% in the remote future choice condition) reported that the immediate shock would hurt them more deeply than the delayed shock. This finding might indicate that the negative utility derived from the outgrowth constitutes a large proportion of the total utility of future experiences. Future research should unpack the two components of the utility of future experiences using ingenious and effective methods and explore how these two types of utilities work. It is likely that these two utilities interact with each other, and the degree to which one of these utilities exerts a stronger impact than the other may depend on various characteristics of the decision situation such as the nature of the negative event, the time delay, the degree of aversion experienced toward the event, and so on.

The present paper was unable to elucidate the causes of individual differences on the timing preference of negative events. Although the majority of participants in these three studies preferred the immediate negative experiences to delayed negative experiences, there were a few people who wanted to postpone the negative event. For example, 17.9% and 26.3% of the participants in Study 3 anticipated more pain from the immediate or near-term negative events than from the more delayed event. These individual differences clearly deserve further investigation, and one potential starting place for this investigation would be examining whether those people who are more likely to anticipate the future preferred the immediate negative event more heavily than did those who are less likely to anticipate the future.

## Supporting Information

S1 Appendix The follow-up investigation in Study 2. (DOCX)
