# Supplementary material for: Pain Now or Later: An Outgrowth Account of Pain-Minimization
Source: PLoS One. 2015 Mar 6;10(3):e0119320. doi: 10.1371/journal.pone.0119320 (PMC4352049; doi:10.1371/journal.pone.0119320)
Supplement: S1 Appendix — (DOCX) [file pone.0119320.s001.docx]

| There are 14 (13, 12, 11, 10, … ,1) days left before the presentation. How do you feel now about the upcoming presentation? Please rate the strength of your feelings in the following scales (1=weak, 5=moderate, 9=strong). | |
| --- | --- |
| I feel happy now. | 1---2---3---4---5---6---7---8---9 |
| I feel anxious now. | 1---2---3---4---5---6---7---8---9 |
| I feel worried now. | 1---2---3---4---5---6---7---8---9 |
| I feel stressed now. | 1---2---3---4---5---6---7---8---9 |
| I feel afraid now. | 1---2---3---4---5---6---7---8---9 |
| I feel angry now. | 1---2---3---4---5---6---7---8---9 |

Note: The participants completed the scale for the last time on the presentation day and the corresponding instruction was read as follows: Today is the presentation day. How do you feel now about the upcoming presentation?
